# Disproportionality evaluation of adverse effects and suicide/self-injury risk factors associated with vortioxetine: a large-scale pharmacovigilance study

**DOI:** 10.3389/fphar.2025.1689634

**Published:** 2025-11-10

**Authors:** Xiangyu Li, Fang Yang, Lingjing Yuan, Li Ma

**Affiliations:** 1 Department of Pharmacy, Shaoxing Keqiao Women and Children΄s Hospital, Shaoxing, Zhejiang, China; 2 Department of Scientific Education, Shaoxing Second Hospital, Shaoxing, Zhejiang, China; 3 Department of Neurology, Shaoxing Keqiao Women and Children΄s Hospital, Shaoxing, Zhejiang, China

**Keywords:** Vortioxetine, adverse drug reaction, FAERS, data mining, pharmacovigilance

## Abstract

**Introduction:**

Adverse events associated with antidepressants may contribute to stigma, morbidity, and suicidal behaviour among patients. Effectively assessing the risk of potential AEs induced by antidepressants is crucial for guiding treatment strategy selection. In this study, the antidepressant-related AEs of vortioxetine and other antidepressants were analysed using data from the FDA Adverse Event Reporting System (FAERS) and the Japanese Adverse Drug Event Report (JADER), with a particular focus on the risk factors for suicide and selfinjury events.

**Methods:**

Demographic data, adverse events types, and frequencies for patients using vortioxetine were summarized from the FDA Adverse Event Reporting System and Japanese Adverse Drug Event Report database, covering the third quarter of 2013 to the third quarter of 2024, through data cleaning, analysis, and statistical methods. The risk signals were identified using four disproportionality methods.

**Results:**

There were 13,097 and 509 AE reports listing vortioxetine as the “primary suspect” from the FAERS and JADER, respectively. Twenty-seven system organ classes and 2002 preferred terms (PTs) signals were identified, and vortioxetine JADER with 25 SOCs (218 PTs). Notable differences existed in the signal strength values obtained for identical PTs from the two databases. More than 20 novel AEs, not listed on the product label, were identified. Notably, stronger correlations for suicide/self-injury were observed for vortioxetine via multivariate logistic regression analysis. Additionally, compared with their male counterparts, female patients are significantly more susceptible to suicide/self-injury. The risk of suicide/self-injury was significantly lower in individuals aged ≥25 years than in those aged 0–24 years (p < 0.05), and the lowest risk was found in patients aged ≥60 years (OR 0.26 (0.22–0.30), p < 0.001).

**Discussion:**

The detected risk signals indicate only the statistical correlation between the target drug and the target adverse reaction and do not indicate the inevitable causal link between the drug and adverse response. Likely confounders include vortioxetine’s preferential prescription in complex patient populations (attributed to its broad-spectrum efficacy and safety profile) rather than intrinsic drug effects. Future studies should consider controlling for these confounders to provide a more definitive assessment.

## 1 Introduction

Depressive disorder is a prevalent mental illness with high prevalence, disease burden, recurrence, disability, and suicide rates. According to the World Health Organization, the COVID-19 pandemic led to a 28% increase in depressive disorders globally in 2020, and it is expected to become the leading cause of disease burden by 2030 (Huang et al., 2019). Depression often persists and can progress to major depressive disorder (MDD), posing treatment challenges. Drug therapy remains the most effective and preferred treatment. Residual cognitive impairment during remission is among the most important factors leading to the recurrence of depression (Saragoussi et al., 2017), and drugs that can improve both depressive mood and cognitive impairment are urgently needed. Vortioxetine is a novel antidepressant with unique properties that targets 5-hydroxytryptamine (5-HT) transporters and various 5-HT receptors. It acts as a partial agonist of 5-HT1A and 5-HT1B and antagonizes the 5-HT1D, 5-HT7, and 5-HT3 receptors (Sanchez et al., 2014). Vortioxetine demonstrates significant efficacy in MDD patients with previous treatment failures, reducing recurrence risk and enhancing social and cognitive functions. Approved by the FDA for MDD treatment on 30 September 2013, vortioxetine has a lower incidence of adverse events than those of traditional antidepressants (tricyclics, monoamine oxidase inhibitors, tetracyclics/heterocyclics, etc.). Studies have indicated that its effectiveness is similar to that of venlafaxine after 8 weeks of treatment (Wang et al., 2015). The 2016 Canadian Network for Mood and Anxiety Treatments guidelines recommend vortioxetine as a first-line treatment alongside level 1 evidence for patients with MDD (Kennedy et al., 2016), which based on positive effects on neuropsychological performance in multiple cognitive domains in patients with MDD. Vortioxetine exhibits a clear dose-response relationship in its therapeutic effects. The agent demonstrates beneficial modulation of cognitive performance and hedonic capacity, thereby substantially enhancing the probability of attaining functional recovery in depressive disorders (Krupa et al., 2023). It is worth noting that the low risk of withdrawal symptoms following discontinuation of vortioxetine treatment (S et al., 2021). In addition, vortioxetine is a drug used in various “difficult” populations–not only in drug-resistant patients but also, for example, in patients diagnosed with epilepsy, bipolar disorder, individuals with somatic diseases, and even elderly patients. (Krupa et al., 2023; Danielak, 2021).

However, in addition to efficacy, adverse drug reactions remain a significant concern for both physicians and patients. Current safety data for vortioxetine are largely derived from clinical trials, which have inherent limitations, such as strict enrolment criteria, small sample sizes, and limited follow-up durations, concentrating AEs in limited organ systems. The FDA Adverse Event Reporting System (FAERS) provides comprehensive global data on vortioxetine-related AEs and serves as a vital tool for real-world safety evaluation. Previous research has validated the FAERS content for targeted analysis (Fang et al., 2023; Shu et al., 2023; Anand et al., 2019). In this study, vortioxetine-related AEs were systematically analysed using the FAERS database, and the differences in frequency and risk ratios related to major organs and systems were evaluated. Our results offer valuable reference information for clinicians and provide comprehensive postmarketing re-evaluation indicators for monitoring and managing vortioxetine in clinical settings.

## 2 Materials and methods

### 2.1 Data sources

FAERS is the world’s largest spontaneously reported AE database, containing AEs, quality complaints leading to AEs, and medication error reports submitted to the FDA from various sources (Wysowski and Swartz, 2005). FDA anonymous data are released to the public and provide opportunities for external researchers and pharmacovigilance experts to explore the data source (Shu et al., 2022). Meanwhile, data were also extracted from the Japanese Adverse Drug Event Report (JADER) databases to verify the results, a spontaneous reporting system managed by the Pharmaceuticals and Medical Devices Agency (PMDA) in Japan. Given that the FAERS and JADER is available to the public, patient records are anonymous; therefore, neither informed consent nor ethical approval is involved. The American Standard Code for Information Interchange (ASCII) files of AE reports from the third quarter of 2013 to the third quarter of 2024 were downloaded from the FAERS database, and data record tables of demographics, drugs, AEs and outcomes were linked using the PRIMARYID key. This time frame unification aims to minimize variability resulting from differences in market availability durations among the studied drugs.

### 2.2 Data processing

**FIGURE 1:**
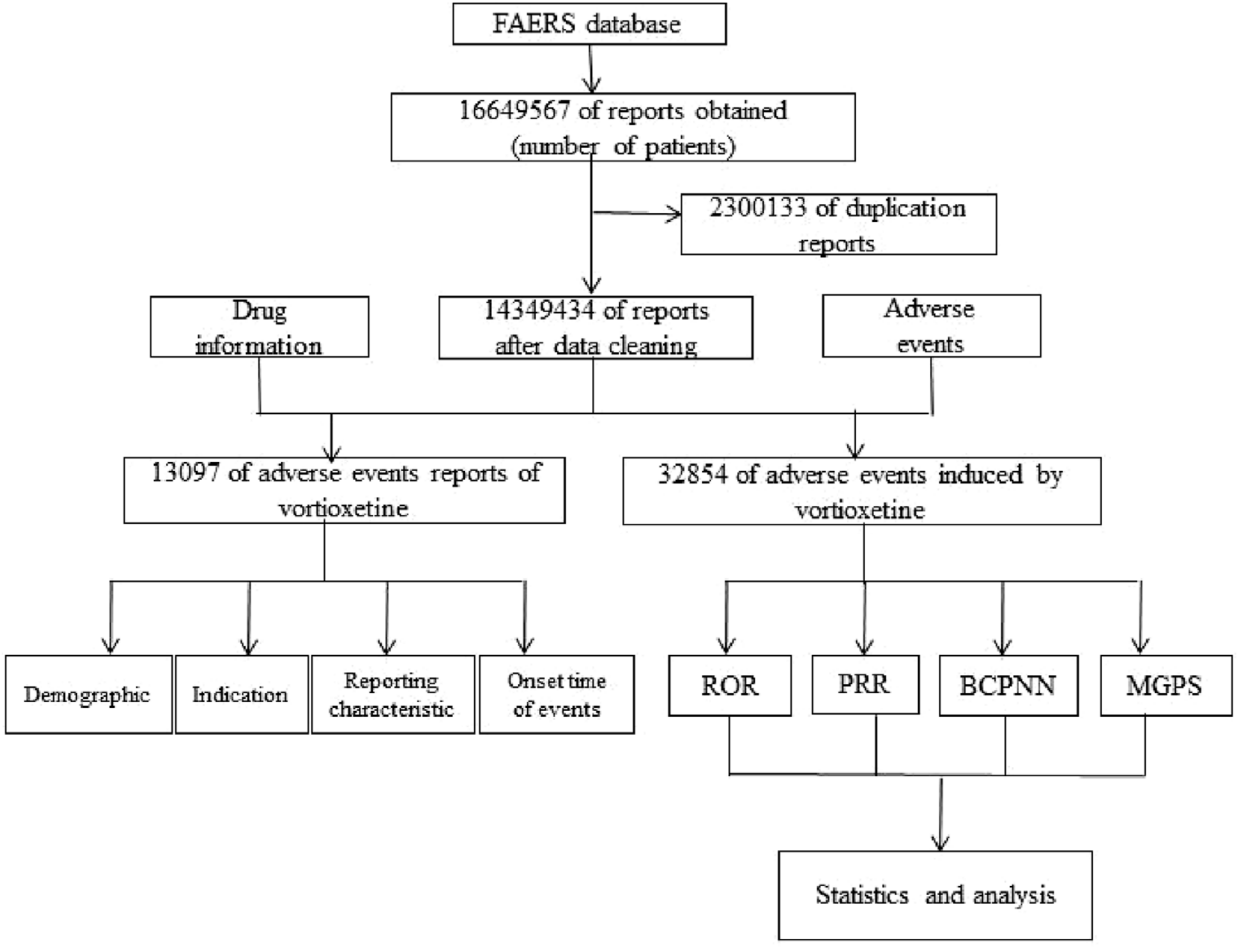
Flow diagram for the selection of adverse events (AE) associated with vortioxetine from FAERS database.

A flow diagram of data extraction and mining is shown in Figure 1. Reports with missing PRIMARYID fields and association errors were removed, and duplicate reports were deleted (the I_F_COD field is F). Reports listing vortioxetine in the PROD_AI field were identified, and those with vortioxetine as the primary suspect (PS) were analysed. The data were imported into R software (version 4.4.1) for data screening and analysis.

The FAERS and JADER databases use preferred terms (PTs) from the Medical Dictionary for Regulatory Activities (MedDRA) to encode AEs. MedDRA version 27.0 was used to classify PTs into system organ classes (SOCs), and the reporting proportions for each SOC were calculated. Statistical analysis was conducted for severe AEs (outcomes such as death, life-threatening conditions, hospitalization, disability, congenital anomalies, or the need for intervention to prevent permanent impairment/damage).

### 2.3 Standard MedDRA query (SMQ) for “suicide/self-injury”

All antidepressants include black box warnings concerning suicidal thoughts and behaviours on their FDA labels. The study not only focuses on identifying post-marketing safety signals related to vortioxetine but also investigates risk factors such as sex, age, and weight associated with vortioxetine-related suicide or self-injury events, and uses bupropion, a widely used antidepressant, as a positive control. Moreover, we compared the top 20 antidepressants in terms of the frequency of suicide/self-injury from the third quarter of 2013 to the third quarter of 2024. To further enhance the credibility of the results, we conducted a stratified analysis of the existing data on vortioxetine according to the daily dose, drug combinations, and indications. Additionally, we conducted sensitivity analyses on vortioxetine and three other antidepressants (duloxetine, venlafaxine, and mirtazapine). The PTs for the SMQ “suicide/self-injury” compiled data on suicidal and self-injurious reports informed by prior studies (Chen et al., 2023). The selected PTs included suspected suicide attempt, suspected suicide, suicide threat, suicide attempt, suicidal ideation, suicidal behaviour, self-injurious ideation, deliberate poisoning, intentional self-injury, intentional overdose, suicidal depression, completed suicide, abnormal Columbia suicide severity rating scale, and assisted suicide. By utilizing recognized and clinically validated terms, the study successfully obtained relevant reports aligning with its objectives.

### 2.4 Statistical analysis

Currently, the primary method for mining AE signals globally is the disproportionality method (Giaquinto et al., 2022), which can reduce the workload of clinical research and focus its scope effectively. Our study utilized four disproportionality methods, namely, the reporting odds ratio (ROR), proportional reporting ratio (PRR), Bayesian confidence propagation neural network (BCPNN), and multi-item gamma Poisson shrinker (MGPS), for data analysis. All disproportionality methods are based on four-grid tables (Supplementary Table S1). The goal of these methods is to calculate the relative frequency of target adverse reactions caused by a specific drug in the FAERS database within a target period. A higher signal intensity (ROR, PRR, IC, and EBGM values) indicates a stronger association between the drug and adverse reactions. The calculation formulas and criteria for each method are provided in Supplementary Table S2. The data were analysed using R software (version 4.4.1) and SPSS software (version 19.0; IBM Corporation). A positive (significant) risk signal must meet the thresholds of all four methods. Furthermore, the stratified analyses were conducted to examine variations based on dosage, concomitant medication use, and therapeutic indications. In addition, We examined suicide/self-injury signals through univariate and multivariate logistic regression analyses to determine the odds ratio (OR) for drug-induced suicide/self-injury under different exposure factors (e.g., sex, age, weight), with *p* < 0.05 indicating significance.

### 2.5 Regression analysis

Only FAERS reports containing complete information on vortioxetine, duloxetine, venlafaxine, and mirtazapine were included. Basic information from these reports included sex, age, weight, and time to onset of suicide/self-injury. First, univariate logistic regression was conducted for suicide/self-injury incidents related to vortioxetine and other antidepressants. Factors with *p* < 0.05 in univariate regression were analysed by multivariate logistic regression. The multivariate logistic regression analysis was applied to assess these risk factors and compare outcomes with other antidepressants, including duloxetine, venlafaxine, and mirtazapine.

## 3 Results

### 3.1 Characteristics of patients treated with vortioxetine

**FIGURE 2:**
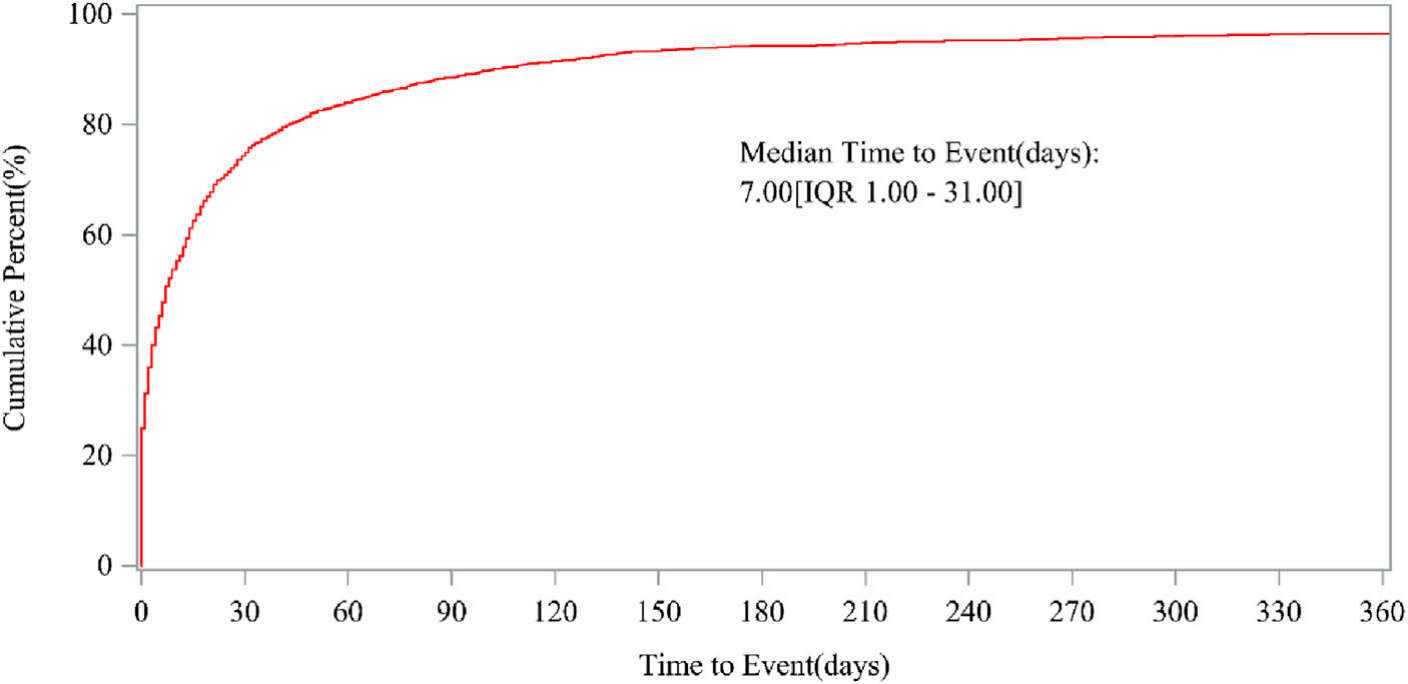
Time to onset of vortioxetine-associated AEs.

As of September 2024, after data cleaning, 13,097 and 509 vortioxetine-related reports were recorded in the FAERS and JADER, respectively. The characteristics are detailed in Table 1 and Supplementary Table S3. Our findings indicate that females constitute 2.3 and 1.6 times more reports than males do, respectively. Compared with the other age groups, the 18–44 (20–40 in JADER) age group appeared to be more susceptible to vortioxetine-related AEs. Vortioxetine-related reports consistently exceeded 1,000 cases annually from 2014 to 2020. The primary reporters were consumers (51.25% vs. 3.5%) and physicians (26.13% vs. 72.7%). The top reporting countries included the United States, Japan, France, Canada, and the United Kingdom. Severe AEs accounted for 38.57% of the reports. Among the AE outcomes, “hospitalization—initial or prolonged” (11.67%) was the most common. The median time to AE onset was 7 days (interquartile range, 1–31) (Figure 2).

**TABLE 1:** Characteristics of all cases treated with vortioxetine from FAERS.

Characteristics	Case number and case proportion (%)
Gender	
Female	7,997 (61.06)
Male	3,470 (26.49)
Not Specified (%)	1,630 (12.45)
Age (years)	
<18 (%)	136 (1.04)
18–44 (%)	2,706 (20.66)
45–64 (%)	2,490 (19.01)
65–74 (%)	721 (5.51)
≥75 (%)	503 (3.84)
Not Specified (%)	6,541 (49.94)
Year of Reports	
2014	1,109 (8.47)
2015	1,433 (10.94)
2016	1,404 (10.72)
2017	1,968 (15.03)
2018	1,570 (11.99)
2019	1,491 (11.38)
2020	1,117 (8.53)
2021	999 (7.63)
2022	849 (6.48)
2023	743 (5.67)
2024	414 (3.16)
Occupation of the reporter	
Consumer	6,712 (51.25)
Not Specified	339 (2.59)
Other health-professional	1,417 (10.82)
Pharmacist	1,203 (9.19)
Physician	3,422 (26.13)
Reporting countries	
United States of America	10,270 (78.41)
Japan	507 (3.87)
France	346 (2.64)
Canada	251 (1.92)
United Kiongdom	181 (1.38)
Serious reportes	
Non-Serious	8,045 (61.43)
Serious	5,052 (38.57)
outcome	
Life-Threatening	203 (1.55)
Hospitalization - Initial or Prolonged	1,529 (11.67)
Disability	218 (1.66)
Death	382 (2.92)
Other	3,726 (28.45)
Time to onset of vortioxetine related AEs (days)	
Median (Q1, Q3)	7.00 (1.00, 31.00)

### 3.2 Descriptive analysis of SOCs and PTs related to vortioxetine

Twenty-seven SOCs and 2002 PTs signals were identified, and vortioxetine-JADER with 25 SOCs (218 PTs). The top three SOCs based on case numbers (Table 2) were psychiatric disorders (n = 7,654, 23.30%), general disorders and administration site conditions (n = 4,835, 14.72%), and gastrointestinal disorders (n = 4,835, 14.72%), which is consistent with the characteristics of vortioxetine as a psychiatric drug. The total number of events in Table 2 is 32,854.

**TABLE 2:** Distribution of adverse events (AE) of vortioxetine in different system organ class (SOC).

System organ class	Case number (n)	Proportion (%)
Psychiatric disorders	7,654	23.30
General disorders and administration site conditions	4,835	14.72
Gastrointestinal disorders	4,835	14.72
Nervous system disorders	4,209	12.81
Injury, poisoning and procedural complications	2,446	7.45
Skin and subcutaneous tissue disorders	1,985	6.04
Investigations	1,328	4.04
Musculoskeletal and connective tissue disorders	701	2.13
Eye disorders	630	1.92
Metabolism and nutrition disorders	580	1.77
Surgical and medical procedures	495	1.51
Respiratory, thoracic and mediastinal disorders	442	1.35
Reproductive system and breast disorders	428	1.3
Cardiac disorders	427	1.3
Infections and infestations	301	0.92
Vascular disorders	285	0.87
Renal and urinary disorders	233	0.71
Ear and labyrinth disorders	182	0.55
Social circumstances	177	0.54
Immune system disorders	138	0.42
Neoplasms benign, malignant and unspecified (incl cysts and polyps)	110	0.33
Pregnancy, puerperium and perinatal conditions	106	0.32
Hepatobiliary disorders	105	0.32
Blood and lymphatic system disorders	90	0.27
Endocrine disorders	80	0.24
Congenital, familial and genetic disorders	28	0.09
Product issues	24	0.07

**FIGURE 3:**
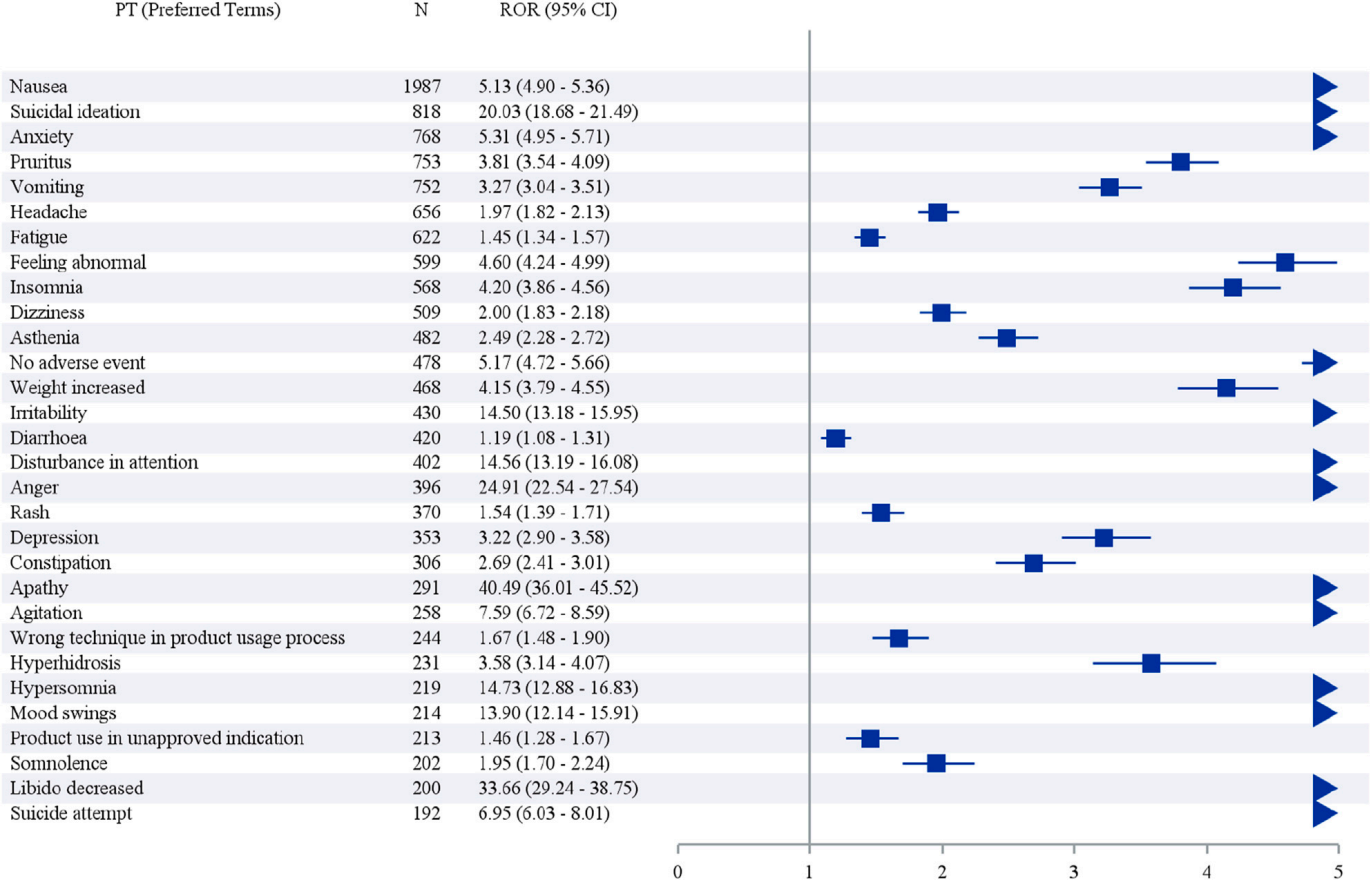
Reporting odds ratio (ROR) analysis of preferred terms with top 30 occurrence frequency of vortioxetine-associated AEs.

**FIGURE 4:**
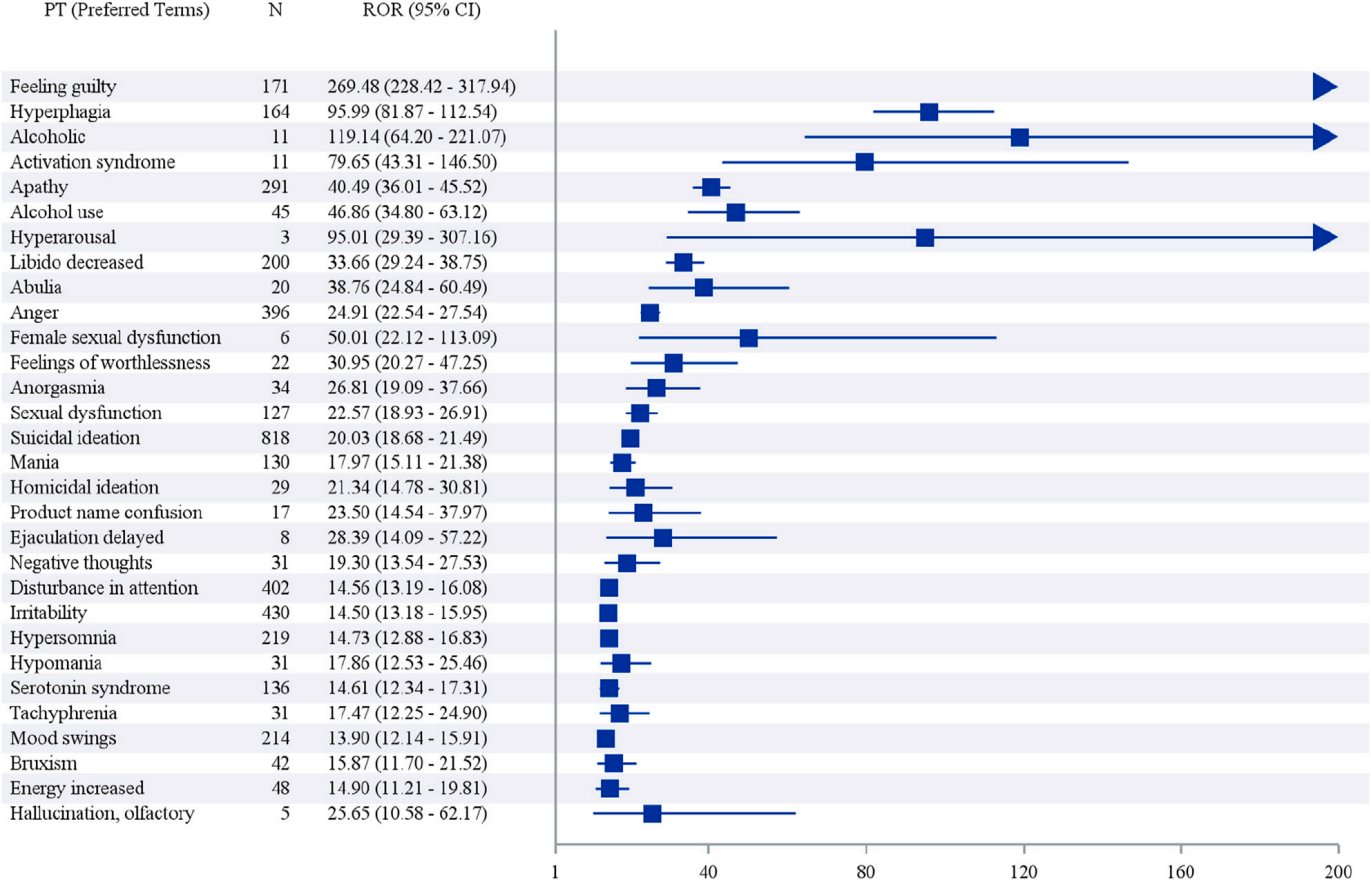
ROR analysis of preferred terms with top 30 positive signal intensity of vortioxetine-associated AEs.

The top 30 vortioxetine-related AE signals by intensity and frequency are displayed in Figures 3, 4 and Tables 3 and 4. Although FAERS includes all health-related AEs, it contains a few non-vortioxetine-induced AE signals likely due to disease progression or other causes, such as product issues. The three most frequent vortioxetine-related AEs were nausea (n = 1,987, ROR 5.13, PRR 4.88, IC 2.28, EBGM 4.86), suicidal ideation (n = 818, ROR 20.03, PRR 19.56, IC 4.27, EBGM 19.28), and anxiety (n = 768, ROR 5.31, PRR 5.21, IC 2.38, EBGM 5.20). Twelve novel AEs were identified that are not listed in the drug package insert: feeling abnormal, insomnia, asthenia, disturbance in attention, apathy, hypersomnia, blurred vision, feeling guilty, decreased libido, hyperphagia, dry mouth, and contusion. Concurrently, the top three AE signals by intensity were feeling guilty (n = 171, ROR 269.48, PRR 268.09, IC 7.79, EBGM 221.44), hyperphagia (n = 164, ROR 95.99, PRR 95.52, IC 6.47, EBGM 88.89), and alcoholism (n = 11, ROR 119.14, PRR 119.10, IC 6.77, EBGM 108.95). Nineteen novel vortioxetine-related AEs not mentioned in the package insert were also found: feelings of guilt, hyperphagia, alcoholism, activation syndrome, apathy, decreased libido, anorgasmia, alcohol use, hyperarousal, abulia, feelings of worthlessness, homicidal ideation, delayed ejaculation, negative thoughts, disturbance in attention, hypersomnia, tachyphrenia, bruxism, and energy. As shown in Supplementary Table S4, The consistent positive signals identified by both FAERS and JADER include suicidal ideation, irritability, apathy, suicide attempt, completed suicide, serotonin syndrome, mania, and activation syndrome.

**TABLE 3:** Disproportionality analysis of preferred terms (PT) with top 30 occurrence frequency of vortioxetine-associated AEs.

SOC	Preferred terms	Case	ROR (95% CI)	PRR (chi-square)	IC (IC-2SD)	EBGM (EBGM05)
Gastrointestinal disorders	Nausea	1,987	5.13 (4.90–5.36)	4.88 (6,175.73)	2.28 (2.21)	4.86 (4.65)
Psychiatric disorders	Suicidal ideation	818	20.03 (18.68–21.49)	19.56 (14,205.3)	4.27 (4.13)	19.28 (17.98)
Psychiatric disorders	Anxiety	768	5.31 (4.95–5.71)	5.21 (2,615.70)	2.38 (2.26)	5.20 (4.84)
Skin and subcutaneous tissue disorders	Pruritus	753	3.81 (3.54–4.09)	3.74 (1,517.11)	1.90 (1.79)	3.73 (3.47)
Gastrointestinal disorders	Vomiting	752	3.27 (3.04–3.51)	3.21 (1,151.83)	1.68 (1.57)	3.21 (2.98)
General disorders and administration site conditions	Feeling abnormal^a^	599	4.60 (4.24–4.99)	4.53 (1,650.30)	2.18 (2.05)	4.52 (4.17)
Psychiatric disorders	Insomnia^a^	568	4.20 (3.86–4.56)	4.14 (1,356.02)	2.05 (1.92)	4.13 (3.80)
General disorders and administration site conditions	Asthenia^a^	482	2.49 (2.28–2.72)	2.47 (422.62)	1.30 (1.16)	2.47 (2.25)
General disorders and administration site conditions	No adverse event	478	5.17 (4.72–5.66)	5.11 (1,576.93)	2.35 (2.20)	5.09 (4.65)
Investigations	Weight increased	468	4.15 (3.79–4.55)	4.10 (1,098.77)	2.03 (1.89)	4.09 (3.74)
Psychiatric disorders	Irritability	430	14.50 (13.18–15.95)	14.32 (5,273.55)	3.83 (3.64)	14.17 (12.88)
Nervous system disorders	Disturbance in attention^a^	402	14.56 (13.19–16.08)	14.40 (4,959.74)	3.83 (3.64)	14.25 (12.91)
Psychiatric disorders	Anger	396	24.91 (22.54–27.54)	24.63 (8,809.22)	4.60 (4.37)	24.18 (21.87)
Psychiatric disorders	Depression	353	3.22 (2.90–3.58)	3.20 (534.29)	1.68 (1.51)	3.19 (2.88)
Gastrointestinal disorders	Constipation	306	2.69 (2.41–3.01)	2.68 (322.07)	1.42 (1.25)	2.67 (2.39)
Psychiatric disorders	Apathy^a^	291	40.49 (36.01–45.52)	40.14 (10,766.7)	5.28 (4.93)	38.94 (34.63)
Psychiatric disorders	Agitation	258	7.59 (6.72–8.59)	7.54 (1,456.88)	2.91 (2.69)	7.50 (6.64)
Skin and subcutaneous tissue disorders	Hyperhidrosis	231	3.58 (3.14–4.07)	3.56 (425.11)	1.83 (1.62)	3.55 (3.12)
Nervous system disorders	Hypersomnia^a^	219	14.73 (12.88–16.83)	14.64 (2,751.65)	3.86 (3.57)	14.48 (12.67)
Psychiatric disorders	Mood swings	214	13.90 (12.14–15.91)	13.81 (2,516.99)	3.77 (3.49)	13.67 (11.95)
Psychiatric disorders	Decreased libido^a^	200	33.66 (29.24–38.75)	33.46 (6,136.81)	5.03 (4.61)	32.62 (28.34)
Psychiatric disorders	Suicide attempt	192	6.95 (6.03–8.01)	6.92 (967.00)	2.78 (2.53)	6.88 (5.97)
Psychiatric disorders	Completed suicide	174	4.55 (3.92–5.28)	4.53 (477.59)	2.18 (1.93)	4.52 (3.89)
Eye disorders	Blurred vision^a^	171	2.52 (2.17–2.93)	2.51 (155.44)	1.33 (1.09)	2.51 (2.16)
Psychiatric disorders	Feeling guilty^a^	171	269.48 (228.42–317.94)	268.09 (37,554.7)	7.79 (6.36)	221.44 (187.69)
Metabolism and nutrition disorders	Hyperphagia^a^	164	95.99 (81.87–112.54)	95.52 (14,263.7)	6.47 (5.62)	88.89 (75.82)
Nervous system disorders	Serotonin syndrome	136	14.61 (12.34–17.31)	14.56 (1,698.03)	3.85 (3.47)	14.40 (12.16)
Gastrointestinal disorders	Dry mouth^a^	133	3.27 (2.76–3.88)	3.26 (208.22)	1.70 (1.43)	3.26 (2.74)
Psychiatric disorders	Mania	130	17.97 (15.11–21.38)	17.91 (2,046.52)	4.14 (3.72)	17.67 (14.86)
Injury, poisoning and procedural complications	Contusion^a^	127	2.49 (2.09–2.96)	2.48 (112.33)	1.31 (1.04)	2.48 (2.08)

Only the PT judged as positive signals were presented.

^a^ AEs, not mentioned in the drug label.

**TABLE 4:** Disproportionality analysis of PTs with top 30 positive signal intensity of vortioxetine-associated AEs.

SOC	Preferred terms	Case	ROR (95% CI)	PRR (chi-square)	IC (IC-2SD)	EBGM (EBGM05)
Psychiatric disorders	Feeling guilty^a^	171	269.48 (228.42–317.94)	268.09 (37,554.7)	7.79 (6.36)	221.44 (187.69)
Metabolism and nutrition disorders	Hyperphagia^a^	164	95.99 (81.87–112.54)	95.52 (14,263.7)	6.47 (5.62)	88.89 (75.82)
Social circumstances	Alcoholism^a^	11	119.14 (64.20–221.07)	119.10 (1,177.45)	6.77 (2.57)	108.95 (58.71)
Psychiatric disorders	Activation syndrome^a^	11	79.65 (43.31–146.50)	79.62 (803.50)	6.23 (2.53)	74.97 (40.76)
Psychiatric disorders	Apathy^a^	291	40.49 (36.01–45.52)	40.14 (10,766.7)	5.28 (4.93)	38.94 (34.63)
Social circumstances	Alcohol use^a^	45	46.86 (34.80–63.12)	46.80 (1,945.20)	5.50 (4.09)	45.17 (33.54)
Psychiatric disorders	Hyperarousal^a^	3	95.01 (29.39–307.16)	95.01 (259.58)	6.47 (0.44)	88.45 (27.36)
Psychiatric disorders	Decreased libido^a^	200	33.66 (29.24–38.75)	33.46 (6,136.81)	5.03 (4.61)	32.62 (28.34)
Psychiatric disorders	Abulia^a^	20	38.76 (24.84–60.49)	38.74 (713.48)	5.23 (3.14)	37.62 (24.11)
Psychiatric disorders	Anger	396	24.91 (22.54–27.54)	24.63 (8,809.22)	4.60 (4.37)	24.18 (21.87)
Reproductive system and breast disorders	Female sexual dysfunction	6	50.01 (22.12–113.09)	50.00 (277.20)	5.59 (1.52)	48.14 (21.29)
Psychiatric disorders	Feelings of worthlessness^a^	22	30.95 (20.27–47.25)	30.93 (622.01)	4.92 (3.12)	30.22 (19.79)
Psychiatric disorders	Anorgasmia^a^	34	26.81 (19.09–37.66)	26.78 (826.50)	4.71 (3.44)	26.25 (18.69)
Reproductive system and breast disorders	Sexual dysfunction	127	22.57 (18.93–26.91)	22.49 (2,562.55)	4.47 (3.99)	22.11 (18.55)
Psychiatric disorders	Suicidal ideation	818	20.03 (18.68–21.49)	19.56 (14,205.3)	4.27 (4.13)	19.28 (17.98)
Psychiatric disorders	Mania	130	17.97 (15.11–21.38)	17.91 (2,046.52)	4.14 (3.72)	17.67 (14.86)
Psychiatric disorders	Homicidal ideation^a^	29	21.34 (14.78–30.81)	21.32 (552.39)	4.39 (3.12)	20.98 (14.54)
Injury, poisoning and procedural complications	Product name confusion	17	23.50 (14.54–37.97)	23.48 (359.30)	4.53 (2.69)	23.07 (14.28)
Reproductive system and breast disorders	Delayed ejaculation^a^	8	28.39 (14.09–57.22)	28.39 (206.74)	4.80 (1.83)	27.79 (13.79)
Psychiatric disorders	Negative thoughts^a^	31	19.30 (13.54–27.53)	19.29 (529.48)	4.25 (3.09)	19.01 (13.33)
Nervous system disorders	Disturbance in attention^a^	402	14.56 (13.19–16.08)	14.40 (4,959.74)	3.83 (3.64)	14.25 (12.91)
Psychiatric disorders	Irritability	430	14.50 (13.18–15.95)	14.32 (5,273.55)	3.83 (3.64)	14.17 (12.88)
Nervous system disorders	Hypersomnia^a^	219	14.73 (12.88–16.83)	14.64 (2,751.65)	3.86 (3.57)	14.48 (12.67)
Psychiatric disorders	Hypomania	31	17.86 (12.53–25.46)	17.84 (486.01)	4.14 (3.02)	17.61 (12.35)
Nervous system disorders	Serotonin syndrome	136	14.61 (12.34–17.31)	14.56 (1,698.03)	3.85 (3.47)	14.40 (12.16)
Psychiatric disorders	Tachyphrenia^a^	31	17.47 (12.25–24.90)	17.45 (474.31)	4.11 (3.00)	17.23 (12.09)
Psychiatric disorders	Mood swings	214	13.90 (12.14–15.91)	13.81 (2,516.99)	3.77 (3.49)	13.67 (11.95)
Psychiatric disorders	Bruxism^a^	42	15.87 (11.70–21.52)	15.85 (577.11)	3.97 (3.10)	15.67 (11.55)
General disorders and administration site conditions	Energy increased^a^	48	14.90 (11.21–19.81)	14.88 (614.19)	3.88 (3.11)	14.72 (11.07)
Psychiatric disorders	Hallucination, olfactory	5	25.65 (10.58–62.17)	25.64 (116.06)	4.65 (1.13)	25.15 (10.38)

This table is sorted in descending order by the lower limit of the 95% confidence interval of ROR. Only the PT judged as positive signals were presented.

^a^ AEs not mentioned in the drug label.

### 3.3 Descriptive analysis for the association of suicide/self-injury with the use of vortioxetine and other antidepressants

**FIGURE 5:**
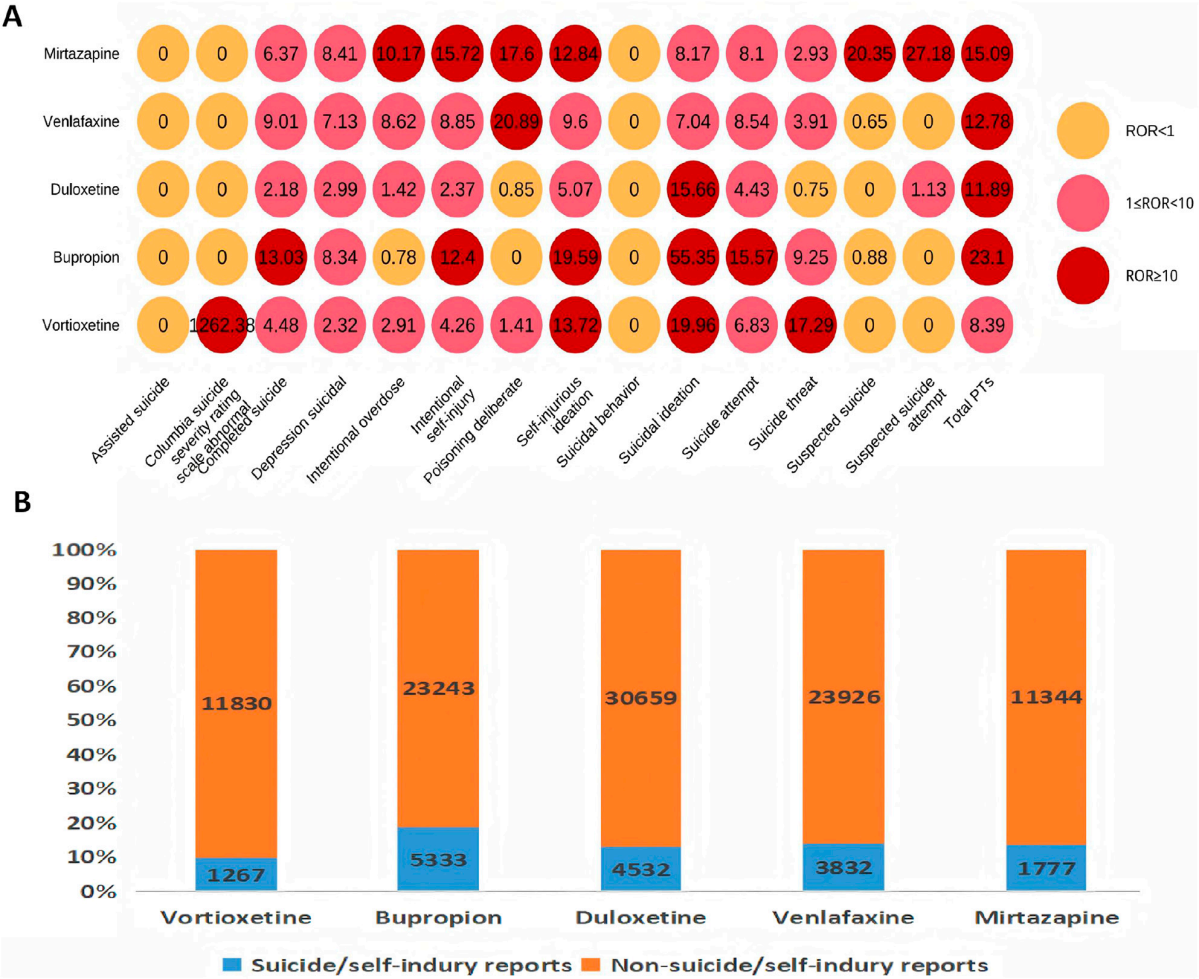
Results of disproportionality analysis for suicidal and self-injurious reports associated with vortioxetine and other antidepressants. **(A)** RORs (95% CI) of antidepressants-associated suicide/self-injury. **(B)** Number of suicide/self-injury and non-suicide/self-injury reports for each drug.

As shown in Supplementary Table S5, bupropion (n = 5,335) had the highest occurrence number, whereas phenelzine (n = 24) had the lowest; according to the disproportionality analysis, doxepin (ROR 23.95) had the highest ROR value, and dextromethorphan/bupropion (ROR 3.66) had the lowest. As shown in Supplementary Table S6, only vortioxetine has “major depression” (18.9%) listed among its top 3 indications. Moreover, the top 3 combined medications it lists are all antidepressants. As shown in Table 5, the top three indications by patient count for those using vortioxetine are depression, major depression, and anxiety. Anxiety is a common comorbidity in patients with depression. Anxiety accounted for 7.0% of vortioxetine indications in our FAERS cohort, which might reflect off-label use despite lacking formal guideline endorsement. The top three antidepressants used in concomitant medication therapy are bupropion, duloxetine, and trazodone. Among these, the concomitant use of trazodone results in a relatively high ROR signal strength for suicide/self-injury events. Stratified analysis by different daily dosages revealed that the >20 mg group demonstrated the highest risk intensity for suicide/self-injury events. Table 6 summarizes the clinical characteristics of suicide/self-injury reports linked to vortioxetine (n = 1,267) and other antidepressants. The number of females surpassed that of males, and the 18–44-year-old age group was the most represented for antidepressants. For most medications, the median time to onset of suicide/self-injury events was less than 1 month. The top 3 suicide/self-injury signals by frequency for these antidepressants were suicidal ideation, suicide attempt, and completed suicide. Figure 5A depicts a significant correlation between suicide/self-injury and vortioxetine [ROR (95% CI): 8.39 (7.92–8.9)] as well as other antidepressants. As shown in Figure 5A, signals (with an ROR 95% CI lower limit >1) were detected for nearly all antidepressants included in the analysis across suicide/self-injury risk events, including completed suicide, suicidal depression, intentional overdose, intentional self-injury, self-injurious ideation, suicidal ideation, suicide attempt, and suicide threat. The strength of the suicide/self-injury risk signals varies across different antidepressants. The proportion of suicide/self-injury AEs associated with vortioxetine (9.6%) was lower than that associated with other antidepressants (Figure 5B).

**TABLE 5:** A stratified analysis of vortioxetine-associated suicide/self-injury reports from FAERS.

Clinical characteristics	Vortioxetine-associated suicide/self-injury reports	ROR analysis
Daily dose		
≤10 mg	635 (77.1%)	8.05 (7.43–8.71)
10–20 mg	140 (17.0%)	7.05 (5.96–8.35)
>20 mg	49 (5.9%)	21.28 (15.85–28.58)
Drug combinations		
Bupropion	32 (4.2%)	7.00 (5.07–9.68)
Duloxetine	26 (3.4%)	9.16 (6.23–13.48)
Trazodone	24 (3.1%)	9.38 (6.68–13.16)
Top three indications		
Depression	475 (37.2%)	—
Major Depression	241 (18.9%)	—
Anxiety	96 (7.0%)	—

**TABLE 6:** Clinical characteristics of vortioxetine and other antidepressants-related suicide/self-injury cases reported to FAERS during the study period.

Clinical characteristics	Vortioxetine-associated suicide/self-injury reports (N = 1,267)	Bupropion-associated suicide/self-injury reports (N = 5,333)	Duloxetine-associated suicide/self-injury reports (N = 4,532)	Venlafaxine-associated suicide/self-injury reports (N = 3,832)	Mirtazapine-associated suicide/self-injury reports (N = 1,777)
Gender					
Female	715 (56.4%)	2,906 (54.5%)	2,758 (60.9%)	2,505 (65.4%)	954 (53.7%)
Male	450 (35.5%)	1,529 (28.7%)	1,146 (25.3%)	993 (25.9%)	696 (39.2%)
Not Specified	102 (8.1%)	898 (16.8%)	628 (13.9%)	334 (8.7%)	127 (7.1%)
Age (years)					
<18	33 (2.6%)	415 (7.8%)	69 (1.5%)	159 (4.1%)	67 (3.8%)
18–44	330 (26.0%)	2,306 (43.2%)	799 (17.6%)	1,647 (43.0%)	564 (31.7%)
45–64	240 (18.9%)	1,356 (25.4%)	787 (17.4%)	1,098 (28.7%)	545 (30.7%)
≥65	74 (4.7%)	260 (4.9%)	198 (4.4%)	316 (8.2%)	324 (18.2%)
Not Specified	590 (46.6%)	996 (18.7%)	2,679 (59.1%)	612 (16.0%)	277 (15.6%)
Outcome					
Death	174 (11.0%)	3,118 (35.1%)	512 (11.3%)	1,208 (31.5%)	476 (26.8%)
Life-Threatening	98 (6.2%)	489 (5.5%)	290 (6.4%)	652 (17.0%)	241 (13.6%)
Hospitalization - Initial or Prolonged	225 (14.3%)	2,247 (25.3%)	597 (13.2%)	1,025 (26.7%)	647 (36.4%)
Disability	23 (1.5%)	54 (0.6%)	73 (1.6%)	74 (1.9%)	40 (2.3%)
Median time to onset of suicide/self-injury	14 d	21 d	78 d	21 d	13 d
PTs of suicide/self-injury					
Assisted suicide	0 (0.0%)	0 (0.0%)	0 (0.0%)	0 (0.0%)	0 (0.0%)
Columbia suicide severity rating scale abnormal	0 (0.0%)	0 (0.0%)	0 (0.0%)	0 (0.0%)	0 (0.0%)
Completed suicide	174 (12.7%)	2,676 (50.2%)	478 (9.2%)	1,123 (24.6%)	357 (16.2%)
Depression suicidal	4 (0.3%)	47 (0.9%)	29 (0.6%)	40 (0.9%)	21 (1.0%)
Intentional overdose	96 (7.0%)	1,293 (24.2%)	269 (5.2%)	926 (20.3%)	487 (22.2%)
Intentional self-injury	52 (3.8%)	90 (1.7%)	164 (3.2%)	350 (7.7%)	275 (12.5%)
Poisoning deliberate	7 (0.5%)	53 (1.0%)	24 (0.5%)	325 (7.1%)	125 (5.7%)
Self-injurious ideation	26 (1.9%)	38 (0.7%)	52 (1.0%)	57 (1.2%)	34 (1.5%)
Suicidal behavior	0 (0.0%)	0 (0.0%)	0 (0.0%)	0 (0.0%)	0 (0.0%)
Suicidal ideation	818 (59.5%)	920 (17.3%)	3,480 (67.0%)	964 (21.1%)	497 (22.6%)
Suicide attempt	192 (14.0%)	601 (11.3%)	693 (13.4%)	771 (16.9%)	327 (14.9%)
Suicide threat	4 (0.3%)	1 (0.02%)	1 (0.0%)	3 (0.1%)	1 (0.0%)
Suspected suicide	0 (0.0%)	312 (5.9%)	0 (0.0%)	5 (0.1%)	68 (3.1%)
Suspected suicide attempt	0 (0.0%)	1 (0.02%)	1 (0.0%)	0 (0.0%)	6 (0.3%)

### 3.4 Factors influencing suicide/self-injury associated with vortioxetine and other antidepressants

**FIGURE 6:**
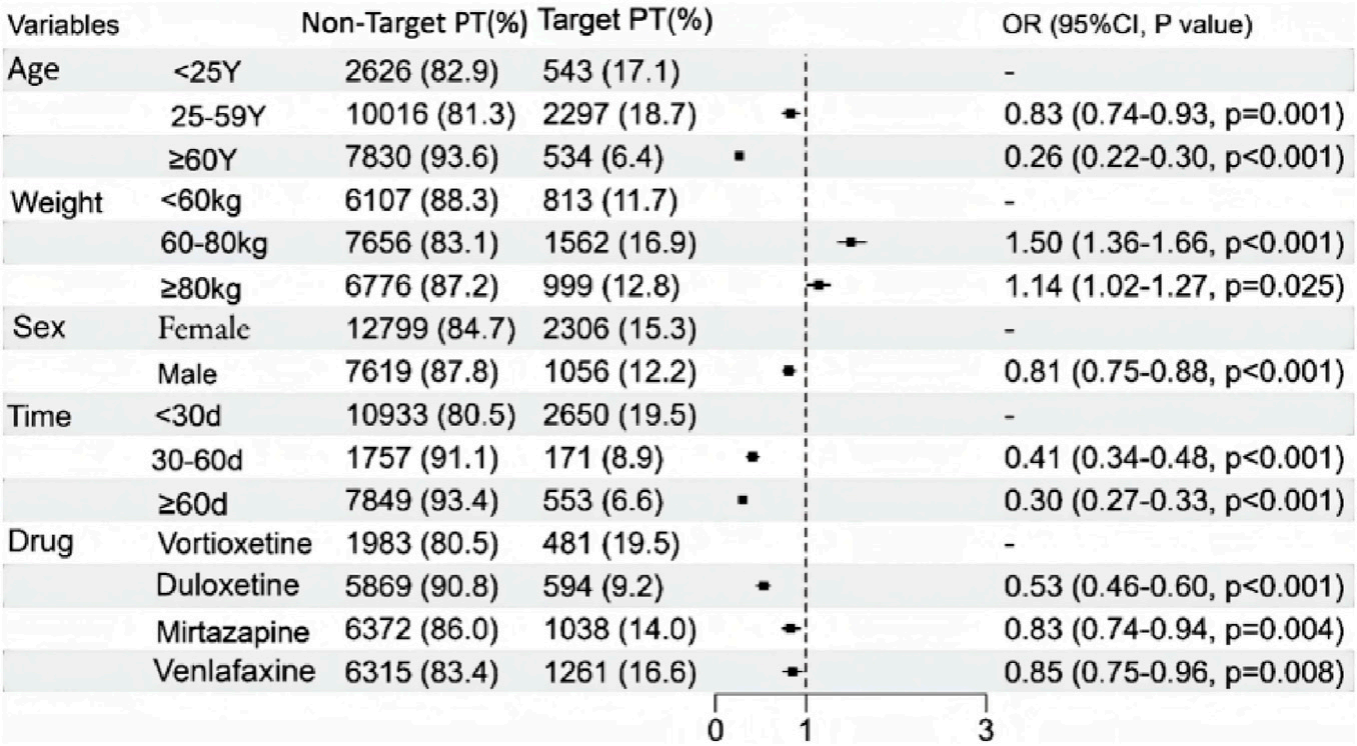
A multivariate logistic regression analysis of conducted on the suicide/self-injury caused by vortioxetine and other antidepressants.

The correlations between patient sex, age, weight, time to onset of adverse events, and drug use and suicide/self-injury are shown in Figure 6. Only reports with complete data were included. Multivariate logistic regression analyses revealed that age, sex, time to onset, drug and weight were significant factors (p < 0.05). The findings of this study revealed that patients under 25 years of age had a significantly greater risk of suicide/self-injury than those aged 25 and above did. Furthermore, the associated risk progressively decreased with increasing age, with the lowest odds ratio (OR) observed in patients older than 60 years [OR 0.26 (0.22–0.30, p < 0.001)]. Additionally, compared with their male counterparts, female patients demonstrated significantly greater susceptibility to treatment-emergent suicide/self-injury. Compared with vortioxetine, duloxetine [OR 0.53 (0.46–0.60, p < 0.001)] was associated with the lowest risk of suicide/self-injury.

## 4 Discussion

To our knowledge, this represents the first analysis of vortioxetine data extracted from both the FAERS and JADER databases. We mined vortioxetine-related data and calculated the ROR, PRR, IC, and EBGM values using four disproportionality methods for AE signal mining. The ROR minimizes bias in fewer events, the PRR offers specificity, the BCPNN effectively cross-validates multisource data, and the MGPS identifies rare events (Lu et al., 2024; Jiang et al., 2024). By exploiting their unique characteristics, this study combines these methods to extend detection and validation from various perspectives. By adjusting the thresholds and variance, this integrated strategy enhances the detection of infrequent responses, reduces false positives *via* cross-validation, and improves safety signal identification accuracy. Unlike previously reported studies (Liu et al., 2025; Li et al., 2025), we not only investigated post-marketing safety signals of vortioxetine but also focused on analysing risk factors associated with vortioxetine-related suicide/self-injury and compared them with those associated with other antidepressants (such as duloxetine, venlafaxine, and mirtazapine).

From this study, we obtained 13,097 reports of vortioxetine-related AEs. The higher incidence in women than in men corresponds to the epidemiology of depression (Zhou et al., 2020). Patients predominantly belonged to the 18–44 age bracket, which is consistent with the target demographic of vortioxetine. This might be related to the higher incidence of MDD in females and/or the 18–44 years age group, which leads to a larger medication base in females and/or this age group and an increase in the absolute number of AEs. While vortioxetine showed off-label use for anxiety (7% of indications), its exclusion from anxiety guidelines underscores a critical gap. Preclinical data suggest 5-HT3 receptor antagonism may confer anxiolytic effects (Sanchez et al., 2014), but clinical trials remain limited. This discordance between real-world practice and guideline recommendations warrants further RCTs to evaluate vortioxetine’s risk-benefit profile in anxiety disorders.

Analysis of the 30 most frequent AEs for vortioxetine revealed that most are listed in the drug package insert, confirming the study’s methodological soundness and reliability of the results. However, in terms of signal strength, many vortioxetine-related adverse reactions not mentioned in the package insert were revealed, indicating the potential presence of novel high-risk signals that need attention in clinical settings. This study revealed that vortioxetine-related AEs were concentrated mainly in patients with psychiatric disorders (n = 7,654). Pharmacological studies have shown that its antidepressant effect is achieved by inhibiting the reuptake of the serotonin transporter, a 5-HT transporter, and regulating 5-HT receptors to increase 5-HT activity in the central nervous system. When the 5-HT system is excessively activated, psychiatric symptoms such as insomnia and anxiety develop. Hence, the susceptibility of patients to psychiatric symptoms associated with vortioxetine is tightly linked with its pharmacology.

Notably, suicidal ideation (n = 818, ROR 20.03, PRR 19.56, IC 4.27, and EBGM 19.28) was the most frequent psychiatric disorder associated with high signal intensity. The FDA required all manufacturers of antidepressants in the United States to include a boxed warning on the package inserts that their use may increase the risk of suicidal thoughts and/or behaviour in children and adolescents in October 2004. In May 2007, the FDA raised the upper age limit for this warning to 25 years and required close monitoring of patients of all ages who use antidepressants. However, no conclusive evidence traces AE occurrence post-antidepressant use in real-world settings. Epidemiologic studies in the United States and various European countries have shown a clear association between increased antidepressant prescriptions and decreased suicide rates (Ludwig and Marcotte, 2005). Depression remains the primary mental illness leading to suicide. While more prescriptions imply effective treatment, possibly reducing suicide rates, antidepressants cannot be directly associated with lower suicide rates. Their therapeutic effects may obscure inherent risks.

Strong correlations for suicide/self-injury were found with the use of vortioxetine and other antidepressants. For most medications, the median time to onset of suicide/self-injury events was less than 1 month. This relates to the mechanism of action of antidepressants, as most antidepressant medications have an onset of effect at 2–4 weeks, whereas a few (such as mirtazapine) may improve certain symptoms within 1–2 weeks, but the full therapeutic efficacy requires more than 4 weeks to evaluate. Additionally, during the early phase of antidepressant treatment, adverse reactions such as anxiety, agitation, and insomnia may occur, potentially exacerbating depressive symptoms. In adolescent populations, there may be an increased risk of suicidal behaviour during initial treatment, which is the basis for the “black box warning” on these medications. This occurs because intense anxiety, in addition to depressive mood, is a significant contributing factor to suicide risk. A meta-analysis of 27 RCTs revealed increased suicide risks with antidepressants for those under 24 years of age, no increased risk for those over 25 years of age, and reduced risks for elderly individuals (Stone et al., 2009). Numerous studies have documented these phenomena, including evidence-based medical research that has conclusively demonstrated the need for clinicians to monitor suicidal tendencies in adolescents when antidepressant medications are prescribed (Friedman and Leon, 2007). However, in this study, the risk of suicide/self-injury was significantly lower among individuals aged ≥25 years than among those aged 0–24 years. There are limitations in evaluating these risks through trials or meta-analyses: (Huang et al., 2019): RCTs are short-term and lack long-term perspectives; (Saragoussi et al., 2017); study populations cannot represent real-world users; and (Sanchez et al., 2014) risk judgement is subjective or study design dependent. Therefore, the factors influencing antidepressant suicide risk and age differences require confirmation *via* prospective studies. Retrospective analysis of FAERS signals (such as time-to-event analysis) combined with subgroup analysis of clinical trials (such as data from adolescents) can support regulatory agencies in formulating risk-minimization strategies (such as revising the black box warning in the package insert). For example, the suicide risk warning for antidepressants in adolescents is based on synergistic evidence from both. The highest risk of adverse events occurring within <30 days may correlate with the onset time of antidepressant efficacy. Additionally, compared with other antidepressants, vortioxetine is associated with the highest risk profile for suicide and self-injurious behaviours according to pharmacovigilance studies. However, the results of controlled studies on vortioxetine, in which various forms of suicidality were extremely rare and no escalation of suicidal ideation was observed (Hochstrasser et al., 2025). We speculate that the following reasons exist. First, the prescribing patterns and treatment histories of patients on vortioxetine may differ significantly from those on other antidepressants. Patients prescribed vortioxetine are often treatment resistant and have failed multiple prior antidepressant treatments. This population may inherently have a higher risk of suicide because of their persistent and severe depressive symptoms (18.9% of the patients were diagnosed with major depression). In contrast, duloxetine is commonly prescribed for depressed patients with chronic pain, a population that may have a lower suicide risk than treatment-resistant patients do. Similarly, mirtazapine and trazodone are often used in low doses for sleep or as adjunctive therapies, which may also be associated with lower suicide/self-injury risks. Second, the timing of the prescription of these medications could play a role. Bupropion, for example, may be prescribed earlier in the treatment course (Clark et al., 2023), whereas vortioxetine is more likely to be prescribed later for patients who have not responded to other therapies. This timing difference could impact the observed suicide/self-injury risk. Furthermore, the pharmacological profiles of these medications may contribute to the varying suicide risks. The mechanism of action of vortioxetine, which involves the modulation of multiple serotonin receptors, may have unique effects on mood and cognition and warrants further investigation. Finally, it is important to consider the possibility of confounding factors, such as comorbid conditions or concurrent medications, that may influence suicide risk. For example, certain types of insomnia have been associated with suicide/self-injury risk (Reffi et al., 2025), which could explain the slightly higher signal observed when combined with trazodone, which is often used for sleep. This finding suggests the urgency of closely monitoring vortioxetine-related suicide/self-injury events. In summary, while maintaining necessary vigilance regarding the potential suicide risk associated with antidepressants, we should not negate their therapeutic efficacy on the basis solely of this concern.

Serotonin syndrome (SS) has emerged because of widespread serotonergic antidepressant use. Although its incidence is low, SS demands clinical attention (Ramachandran et al., 2018; Little et al., 2018). Reports have shown that SS also occurs with other drugs that may affect serum 5-HT levels (Sweileh, 2024). SS showed a low-frequency but significant risk signal (n = 136; ROR, 14.61; PRR, 14.56; IC, 3.85; and EBGM, 14.40). Given the contribution of signal intensity from database events and vortioxetine-related AEs, low n values but high-intensity results warrant further verification. SS mainly presents as anxiety and agitation; it can escalate to confusion and autonomic symptoms when it is untreated and is potentially fatal. Hence, early SS detection is critical.

More than 20 novel AE risk signals in this study were not documented in drug instructions. These symptoms can lead to a decrease in medication compliance among patients, thus affecting treatment efficacy. Among the above symptoms, the most easily ignored risk signal with a high signal intensity is decreased libido (n = 200, ROR 33.66, PRR 33.46, IC 5.03, and EBGM 32.62), which is related to sexual dysfunction, an adverse reaction recorded in the instructions. Previous studies revealed that the incidence of AEs related to sexual dysfunction ranged from 1.6% to 1.8% in the vortioxetine group, compared with 1.0% in the placebo group; the incidence in the vortioxetine female group ranged from 0.6% to 1.1%, and that in the vortioxetine male group ranged from 2.8% to 3.6% (Baldwin et al., 2016), which suggested that the risk among males was greater than that among females. Another study revealed that the incidence of sexual dysfunction was 2.0% with 15 mg of vortioxetine and 4.0% with 20 mg of vortioxetine, suggesting that the incidence of sexual dysfunction increased with increasing drug dose (Boulenger et al., 2014). In addition to the adverse reactions included in the instructions, we should pay attention to the positive risk signals not mentioned in the instructions when using vortioxetine in clinical practice.

However, this study has several limitations. FAERS and JADER are spontaneous submission database. Owing to the differences in the quality of the reporting personnel, there is a certain bias in the quality and completeness of the reported data. Calculating incidence rates is not possible with the available data. In addition, the data obtained in the study could only be queried for the results but not for the original data; thus, analysing related factors such as the dose of medication and the course of treatment was not possible, resulting in a certain bias in the analysis results. The detected risk signals indicate only the statistical correlation between the target drug and the target adverse reaction and do not indicate the inevitable causal link between the drug and adverse response. There is a high probability that the obtained results are a result of vortioxetine’s frequent use in particularly difficult and problematic populations (due to its broad profile of action, safety, and efficacy), rather than the pharmacological properties and effects of the drug. Therefore, the role of the vortioxetine-related risk signal requires further clinical drug observation to be evaluated and validated. Future studies should consider controlling for treatment resistance, the timing of medication initiation, and other confounding factors to provide a more definitive assessment.

## 5 Conclusion

In conclusion, this study adds to real-world safety data concerning vortioxetine, stressing the importance for clinicians to monitor associated adverse reactions vigilantly. Compared with other antidepressants, vortioxetine results in stronger suicide and self-injury risk possibly linked to treatment-resistant cases, difficoult–to–treat and other problematic subpopulations with comorbidities. Young adults/females should be monitored closely during initial treatment (median time to onset: 7 days). Future pharmaco-epidemiological studies should explore potential connections between vortioxetine-related suicide/self-injury and patient age.

## Data Availability

The original contributions presented in the study are included in the article/Supplementary Material, further inquiries can be directed to the corresponding authors.

## References

[B1] Anand K. Ensor J. Trachtenberg B. Bernicker E. H. (2019). Osimertinib-induced cardiotoxicity: a retrospective review of the FDA adverse events Reporting system (FAERS). JACC Cardio Oncol. 1, 172–178. 10.1016/j.jaccao.2019.10.006 34396179 PMC8352117

[B2] Baldwin D. S. Chrones L. Florea I. Nielsen R. Nomikos G. G. Palo W. (2016). The safety and tolerability of vortioxetine: analysis of data from randomized placebo-controlled trials and open-label extension studies. J. Psychopharmacol. 30, 242–252. 10.1177/0269881116628440 26864543 PMC4794082

[B3] Boulenger J.-P. Loft H. Olsen C. K. (2014). Efficacy and safety of vortioxetine (Lu AA21004), 15 and 20 mg/day: a randomized, double-blind, placebo-controlled, duloxetine-referenced study in the acute treatment of adult patients with major depressive disorder. Int. Clin. Psychopharmacol. 29, 138–149. 10.1097/YIC.0000000000000018 24257717 PMC3979887

[B4] Chen C. Zhou R. Fu F. Xiao J. (2023). Postmarket safety profile of suicide/self-injury for GLP-1 receptor agonist: a real-world pharmacovigilance analysis. Eur. Psychiatry 66, e99. 10.1192/j.eurpsy.2023.2474 38031404 PMC10755578

[B5] Clark A. Tate B. Urban B. Schroeder R. Gennuso S. Ahmadzadeh S. (2023). Bupropion mediated effects on depression, attention deficit hyperactivity disorder, and smoking cessation. Health Psychol. Res. 11, 81043. 10.52965/001c.81043 37405312 PMC10317506

[B6] Danielak D. (2021). Vortioxetine in management of major depressive disorder - a favorable alternative for elderly patients? Expert Opin. Pharmacother. 22 (9), 1167–1177. 10.1080/14656566.2021.1880567 33650935

[B7] Fang Z. Xu Z. Zhu W. Yu M. Ji C. (2023). A real-world disproportionality analysis of apalutamide: data mining of the FDA adverse event reporting system. Front. Pharmacol. 14, 1101861. 10.3389/fphar.2023.1101861 37342589 PMC10277739

[B8] Friedman R. A. Leon A. C. (2007). Expanding the black box - depression, antidepressants, and the risk of suicide. N. Engl. J. Med. 356, 2343–2346. 10.1056/NEJMp078015 17485726

[B9] Giaquinto A. N. Sung H. Miller K. D. Kramer J. L. Newman L. A. Minihan A. (2022). Breast cancer statistics. CA Cancer J. Clin. 72, 524–541. 10.3322/caac.21754 36190501

[B10] Hochstrasser B. Hasler G. Baumann A. Bose R. Reines E. Kammerer M. (2025). Effectiveness and tolerability of vortioxetine oral drops *versus* oral tablets in major depressive disorder: an analysis of a real-world cohort study in Switzerland. CNS Drugs 39 (10), 1047–1059. 10.1007/s40263-025-01207-2 40717157 PMC12423148

[B11] Huang Y. Wang Y. Wang H. Liu Z. Yu X. Yan J. (2019). Prevalence of mental disorders in China: a cross-sectional epidemiological study. Lancet Psychiatry 6, 211–224. 10.1016/S2215-0366(18)30511-X 30792114

[B12] Jiang Y. Cheng Y. Du Z. Shen Y. Zhou Q. Ji Y. Zhu H. (2024). Unveiling potential adverse events associated with escitalopram oxalate: a real-world analysis based FDA adverse event reporting system database. J. Psychopharmacol. 38, 567–578. 10.1177/02698811241249651 38678377

[B13] Kennedy S. H. Lam R. W. McIntyre R. S. Tourjman S. V. Bhat V. Blier P. (2016). Canadian network for mood and anxiety treatments (CANMAT) 2016 clinical guidelines for the management of adults with major depressive disorder: section 3. Pharmacological treatments. Can. J. Psychiatry 61, 540–560. 10.1177/0706743716659417 27486148 PMC4994790

[B14] Krupa A. J. Wojtasik-Bakalarz K. Siwek M. (2023). Vortioxetine - pharmacological properties and use in mood disorders. The current state of knowledge. Psychiatr. Pol. 57 (6), 1109–1126. 10.12740/PP/OnlineFirst/151570 36571300

[B15] Li L. Xu Q. Pang L. Liu Y. Lu Y. (2025). Comprehensive analysis of adverse events associated with vortioxetine using the FDA adverse event reporting system. Front. Pharmacol. 16, 1519865. 10.3389/fphar.2025.1519865 40385485 PMC12081438

[B16] Little K. Lin C. M. Reynolds P. M. (2018). Delayed serotonin syndrome in the setting of a mixed fluoxetine and serotonin antagonist overdose. Am. J. Case Rep. 19, 604–607. 10.12659/AJCR.909063 29795058 PMC5994973

[B17] Liu M. Xia Y. Long W. Han X. Xiong Y. (2025). A pharmacovigilance study of vortioxetine based on data from the FDA adverse event reporting system. Sci. Rep. 15 (1), 28886. 10.1038/s41598-025-13786-7 40775011 PMC12332032

[B18] Lu R. Jiang Y. Du Z. Zhou Q. Shen Y. Zhu H. (2024). Multidimensional assessment of adverse events of bupropion: a large-scale data analysis from the FAERS database. J. Affect Disord. 354, 649–655. 10.1016/j.jad.2024.03.085 38494134

[B19] Ludwig J. Marcotte D. E. (2005). Anti-depressants, suicide, and drug regulation. J. Policy Anal. Manage 24, 249–272. 10.1002/pam.20089 15776534

[B20] Ramachandran V. Ding B. George R. Novakovic M. (2018). Conservative management of severe serotonin syndrome with coma, myoclonus, and crossed-extensor reflex complicated by hepatic encephalopathy. Proc. (Bayl Univ. Med. Cent.) 31, 112–114. 10.1080/08998280.2017.1400874 29686575 PMC5903527

[B21] Reffi A. N. Kalmbach D. A. Cheng P. Moore D. A. Jennings M. B. Mahr G. C. (2025). Nightmares and insomnia within the acute aftermath of trauma prospectively predict suicidal ideation. J. Clin. Sleep. Med. 21 (9), 1519–1527. 10.5664/jcsm.11744 40265245 PMC12406830

[B22] Siwek M. Chrobak A. A. Gorostowicz A. Krupa A. J. Dudek D. (2021). Withdrawal symptoms following discontinuation of vortioxetine-retrospective chart review. Pharm. (Basel) 14 (5), 451. 10.3390/ph14050451 34064611 PMC8151377

[B23] Sanchez C. Asin K. E. Vortioxetine A. F. (2014). Vortioxetine, a novel antidepressant with multimodal activity: review of preclinical and clinical data. Pharmacol. Ther. 145, 43–57. 10.1016/j.pharmthera.2014.07.001 25016186

[B24] Saragoussi D. Touya M. Haro J. M. Jönsson B. Knapp M. Botrel B. (2017). Factors associated with failure to achieve remission and with relapse after remission in patients with major depressive disorder in the PERFORM study. Neuropsychiatr. Dis. Treat. 13, 2151–2165. 10.2147/NDT.S136343 28860772 PMC5558880

[B25] Shu Y. He X. Liu Y. Wu P. Zhang Q. (2022). A real-world disproportionality analysis of olaparib: data mining of the public version of FDA adverse event reporting system. Clin. Epidemiol. 14, 789–802. 10.2147/CLEP.S365513 35789689 PMC9250344

[B26] Shu Y. Ding Y. He X. Liu Y. Wu P. Zhang Q. (2023). Hematological toxicities in PARP inhibitors: a real-world study using FDA adverse event reporting system (FAERS) database. Cancer Med. 12, 3365–3375. 10.1002/cam4.5062 35871395 PMC9939145

[B27] Stone M. Laughren T. Jones M. L. Levenson M. Holland P. C. Hughes A. (2009). Risk of suicidality in clinical trials of antidepressants in adults: analysis of proprietary data submitted to US Food and Drug Administration. BMJ 339, b2880. 10.1136/bmj.b2880 19671933 PMC2725270

[B28] Sweileh W. M. (2024). Neuroleptic malignant syndrome and serotonin syndrome: a comparative bibliometric analysis. Orphanet J. Rare Dis. 19, 221. 10.1186/s13023-024-03227-5 38825678 PMC11145872

[B29] Wang G. Gislum M. Filippov G. Montgomery S. (2015). Comparison of vortioxetine *versus* venlafaxine XR in adults in Asia with major depressive disorder: a randomized, double-blind study. Curr. Med. Res. Opin. 31, 785–794. 10.1185/03007995.2015.1014028 25650503

[B30] Wysowski D. K. Swartz L. (2005). Adverse drug event surveillance and drug withdrawals in the United States, 1969–2002: the importance of reporting suspected reactions. Arch. Intern Med. 165, 1363–1369. 10.1001/archinte.165.12.1363 15983284

[B31] Zhou J. Yuan X. Qi H. Liu R. Li Y. Huang H. (2020). Prevalence of depression and its correlative factors among female adolescents in China during the coronavirus disease 2019 outbreak. Glob. Health 16, 69. 10.1186/s12992-020-00601-3 32723373 PMC7385712

