# Supplementary material for: Disproportionality evaluation of adverse effects and suicide/self-injury risk factors associated with vortioxetine: a large-scale pharmacovigilance study
Source: Front Pharmacol. 2025 Nov 10;16:1689634. doi: 10.3389/fphar.2025.1689634 (PMC12640903; doi:10.3389/fphar.2025.1689634)
Supplement: Supplementary file 2 [file Supplementaryfile1.docx]

**Table S1.** Four-grid Table of disproportionality analysis.

|  | Target AE | Other AEs | Total |
| --- | --- | --- | --- |
| Target drug | a | b | a+b |
| Other drugs | c | d | c+d |
| Total | a+c | b+d | N=a+b+c+d |

**Table S2.** Four major algorithms used for signal detection.

| Algorithms | Calculation formulas | Criteria |
| --- | --- | --- |
| ROR | ROR＝(a/c)/(b/d) | 95%CI > 1, a≥3 |
|  | 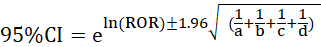 |  |
| PRR | PRR＝[a/(a+b)]/[c/(c+d)] | PRR≥2, χ2 ≥ 4, a≥3 |
|  | *χ*2＝(ad-bc)**^2^**(a+b+c+d)/[(a+b) (c+d) (a+c) (b+d)] |  |
| BCPNN | IC=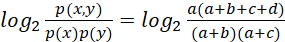 | IC-2SD*>0* |
|  | IC-2SD*=E(IC)-2*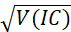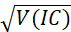 |  |
| MGPS | 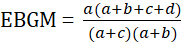 | EBGM05 > 2 |
|  | 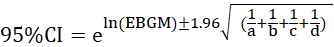 |  |

**Table S3.** Characteristics of all cases treated with vortioxetine from JADER

| Characteristics | Case number and case proportion (%) |
| --- | --- |
| Total | 509 |
| Gender |  |
| Female | 301 (59.1%) |
| Male | 186 (36.5%) |
| Not Specified | 22 (4.3%) |
| Age |  |
| <20 | 21 (4.1%) |
| 20-40 | 198 (38.8%) |
| 50-60 | 101 (19.8%) |
| ≥70 | 119 (23.3%) |
| Not Specified | 70 (13.7%) |
| Year of Reports |  |
| 2019 | 16 (3.14%) |
| 2020 | 77 (15.1%) |
| 2021 | 143 (28.0%) |
| 2022 | 108 (21.2%) |
| 2023 | 91 (17.8%) |
| 2024 | 74 (14.5%) |
| Occupation of the reporter |  |
| Consumer | 18 (3.5%) |
| Other health-professional | 62 (12.1%) |
| Pharmacist | 59 (11.6%) |
| Physician | 370 (72.7%) |
| Outcome |  |
| Recovery | 243 (47.7%) |
| Mild recovery | 96 (18.8%) |
| Death | 39 (7.66%) |
| Not yet recovered | 39 (7.66%) |

**Table S4.** Disproportionality analysis of vortioxetine-associated adverse events in JADER, consistent with that (the top 30 occurrence frequency and intensity positive signal ) in FAERS

| SOC | Preferred Terms | Case | ROR(95% CI) | PRR(Chi-Square) | IC(IC-2SD) | EBGM(EBGM05) |
| --- | --- | --- | --- | --- | --- | --- |
| Gastrointestinal disorders | Nausea | 6 | 1.39 ( 0.62 - 3.1 ) | 1.38 ( 0.64 ) | 0.47 ( -1.2 ) | 1.38 ( 0.62 ) |
| Psychiatric disorders | Suicidal ideation | 41 | 95.23 ( 68.9 - 131.62 ) | 89.52 ( 3409.23 ) | 6.41 ( 4.74 ) | 85.03 ( 61.52 ) |
| Psychiatric disorders | Anxiety | 1 | 3.64 ( 0.51 - 25.97 ) | 3.64 ( 1.91 ) | 1.86 ( 0.19 ) | 3.64 ( 0.51 ) |
| Skin and subcutaneous tissue disorders | Pruritus | 1 | 0.51 ( 0.07 - 3.6 ) | 0.51 ( 0.48 ) | -0.98 ( -2.65 ) | 0.51 ( 0.07 ) |
| Gastrointestinal disorders | Vomiting | 5 | 1.55 ( 0.64 - 3.75 ) | 1.55 ( 0.98 ) | 0.63 ( -1.04 ) | 1.55 ( 0.64 ) |
| Psychiatric disorders | Insomnia* | 1 | 2.23 ( 0.31 - 15.87 ) | 2.23 ( 0.68 ) | 1.15 ( -0.52 ) | 2.23 ( 0.31 ) |
| Psychiatric disorders | Irritability | 3 | 17.1 ( 5.47 - 53.47 ) | 17.03 ( 44.83 ) | 4.08 ( 2.4 ) | 16.87 ( 5.4 ) |
| Psychiatric disorders | Depression | 7 | 19.57 ( 9.25 - 41.38 ) | 19.38 ( 120.67 ) | 4.26 ( 2.59 ) | 19.17 ( 9.06 ) |
| Psychiatric disorders | Apathy* | 1 | 35.68 ( 4.92 - 259.01 ) | 35.63 ( 32.96 ) | 5.13 ( 3.41 ) | 34.91 ( 4.81 ) |
| Psychiatric disorders | Suicide attempt | 33 | 37.26 ( 26.17 - 53.06 ) | 35.5 ( 1084.84 ) | 5.12 ( 3.45 ) | 34.78 ( 24.43 ) |
| Psychiatric disorders | Completed suicide | 20 | 57.4 ( 36.52 - 90.2 ) | 55.73 ( 1040.94 ) | 5.75 ( 4.08 ) | 53.97 ( 34.34 ) |
| Nervous system disorders | Serotonin syndrome | 38 | 100.42 ( 71.77 - 140.52 ) | 94.84 ( 3341.44 ) | 6.49 ( 4.82 ) | 89.81 ( 64.19 ) |
| Psychiatric disorders | Mania | 4 | 24.5 ( 9.1 - 65.93 ) | 24.36 ( 88.34 ) | 4.59 ( 2.91 ) | 24.02 ( 8.93 ) |
| Injury, poisoning and procedural complications | Contusion* | 1 | 3.84 ( 0.54 - 27.34 ) | 3.83 ( 2.09 ) | 1.94 ( 0.26 ) | 3.83 ( 0.54 ) |
| Psychiatric disorders | Activation syndrome* | 4 | 97.66 ( 35.54 - 268.35 ) | 97.09 ( 359.57 ) | 6.52 ( 4.82 ) | 91.82 ( 33.42 ) |

**Table S5.** Disproportionality analysis of top 20 antidepressants-related suicide/self-injury cases from FAERS

| Drug | Case number | ROR (95% CI) |
| --- | --- | --- |
| Bupropion | 5335 | 23.1(22.33-23.89) |
| Duloxetine | 4532 | 11.89(11.52-12.27) |
| Sertraline | 4187 | 10.21(9.88-10.55) |
| Venlafaxine | 3832 | 12.78(12.35-13.23) |
| Fluoxetine | 2838 | 15.31(14.7-15.94) |
| Citalopram | 2443 | 13.91(13.32-14.52) |
| Escitalopram | 1850 | 11.1(10.57-11.66) |
| Mirtazapine | 1768 | 12.28(11.68-12.92) |
| Paroxetine | 1573 | 11.63(11.03-12.27) |
| Vortioxetine | 1255 | 8.39(7.92-8.9) |
| Esketamine | 1254 | 14.07(13.25-14.95) |
| Trazodone | 645 | 15.09(13.87-16.42) |
| Amitriptyline | 607 | 14.72(13.5-16.06) |
| Desvenlafaxine | 509 | 5.3(4.84-5.8) |
| Doxepin | 346 | 23.95(21.23-27.01) |
| Clomipramine | 110 | 12.28(10.04-15.01) |
| Fluvoxamine | 110 | 13.36(10.92-16.36) |
| Dextromethorphan/Bupropion | 44 | 3.66(2.7-4.95) |
| Imipramine | 34 | 7.07(4.97-10.04) |
| Phenelzine | 24 | 4.09(2.71-6.16) |

**Table S6.** A stratified analysis of vortioxetine and other antidepressants-associated suicide/self-injury reports from FAERS

| Antidepressants-associated suicide/self-injury reports | Vortioxetine | Duloxetine | Venlafaxine | Mirtazapine |
| --- | --- | --- | --- | --- |
| **Daily dose** |  |  |  |  |
| Low dose | ≤10mg(635,77.1%) | ≤30mg(504,37.78%) | ≤75mg(321,33.5%) | ≤15mg (210,36.6%) |
| Medium dose | 10-20mg(140,17.0%) | 30-60mg(614,46.03%) | 75-225mg(349,36.4%) | 15-45mg(191,33.3%) |
| High dose | >20mg(49,5.9%) | >60mg(216,16.19%) | >225mg(289,30.1%) | >45mg(173,30.1%) |
| **The top three most frequently combined medications** |  |  |  |  |
| First | Bupropion(32,4.2%) | Alprazolam(105,1.5%) | ‌Quetiapine(246,1.5%) | Zopiclone‌(89,1.7%) |
| Second | Duloxetine(26,3.4%) | Pregabalin‌(104,1.5%) | Zopiclone‌(111,1.5%) | ‌Diazepam‌(77,1.4%) |
| Third | Trazodone(24,3.1%) | Trazodone‌(99,1.4%) | Lamotrigine(110,1.5%) | Citalopram(70,1.3%) |
| **The top three indications** |  |  |  |  |
| First | Depression(475,37.2%) | Depression(1559,35.6%) | Depression(749,28.9%) | Depression(423,28.9%) |
| Second | Major Depression(241,18.9%) | Pain(777,17.8%) | Anxiety(228,8.8%) | Anxiety(129,8.5%) |
| Third | Anxiety(96,7.0%) | Anxiety(339,7.7%) | Suicide Attempt(129,5.0%) | Insomnia(102,6.7%) |
